# Prognosis and Personalized In Silico Prediction of Treatment Efficacy in Cardiovascular and Chronic Kidney Disease: A Proof-of-Concept Study

**DOI:** 10.3390/ph16091298

**Published:** 2023-09-14

**Authors:** Mayra Alejandra Jaimes Campos, Iván Andújar, Felix Keller, Gert Mayer, Peter Rossing, Jan A. Staessen, Christian Delles, Joachim Beige, Griet Glorieux, Andrew L. Clark, William Mullen, Joost P. Schanstra, Antonia Vlahou, Kasper Rossing, Karlheinz Peter, Alberto Ortiz, Archie Campbell, Frederik Persson, Agnieszka Latosinska, Harald Mischak, Justyna Siwy, Joachim Jankowski

**Affiliations:** 1 Mosaiques Diagnostics GmbH, 30659 Hannover, Germany; jaimes@mosaiques-diagnostics.com (M.A.J.C.); latosinska@mosaiques-diagnostics.com (A.L.); mischak@mosaiques-diagnostics.com (H.M.); siwy@mosaiques-diagnostics.com (J.S.); 2 Institute for Molecular Cardiovascular Research, University Hospital RWTH Aachen, 52074 Aachen, Germany; 3 Proteomic Laboratory, Center for Genetic Engineering and Biotechnology, Havana 10600, Cuba; 4 Department of Internal Medicine IV (Nephrology and Hypertension), Medical University Innsbruck, 6020 Innsbruck, Austria; felix.keller@i-med.ac.at (F.K.); gert.mayer@i-med.ac.at (G.M.); 5 Steno Diabetes Center Copenhagen, 2730 Herlev, Denmark; peter.rossing@regionh.dk (P.R.); frederik.persson@regionh.dk (F.P.); 6 Department of Clinical Medicine, University of Copenhagen, 2200 Copenhagen, Denmark; kasper.rossing@regionh.dk; 7 Non-Profit Research Institute Alliance for the Promotion of Preventive Medicine, 2800 Mechlin, Belgium; jan.staessen@appremed.org; 8 School of Cardiovascular and Metabolic Health, University of Glasgow, Glasgow G12 8TA, UK; christian.delles@glasgow.ac.uk (C.D.); william.mullen@glasgow.ac.uk (W.M.); 9 Division of Nephrology and KfH Renal Unit, Hospital St Georg, 04129 Leipzig, Germany; joachim.beige@kfh.de; 10 Medical Clinic 2, Martin-Luther-University Halle/Wittenberg, 06112 Halle, Germany; 11 Nephrology Section, Department of Internal Medicine, Ghent University Hospital, 9000 Ghent, Belgium; griet.glorieux@ugent.be; 12 Hull University Teaching Hospitals NHS Trust, Castle Hill Hospital, Cottingham HU16 5JQ, UK; a.l.clark@hull.ac.uk; 13 Institut National de la Santé et de la Recherche Médicale, Institute of Cardiovascular and Metabolic Disease, UMRS 1297, 31432 Toulouse, France; joost-peter.schanstra@inserm.fr; 14 Renal Fibrosis, Université Toulouse III Paul-Sabatier, Route de Narbonne, 31062 Toulouse, France; 15 Centre of Systems Biology, Biomedical Research Foundation of the Academy of Athens (BRFAA), 115 27 Athens, Greece; vlahoua@bioacademy.gr; 16 Department of Cardiology, Rigshospitalet, Copenhagen University Hospital, 2100 Copenhagen, Denmark; 17 Atherothrombosis and Vascular Biology Program, Baker Heart and Diabetes Institute, 75 Commercial Road, Melbourne, VIC 3004, Australia; karlheinz.peter@baker.edu.au; 18 Department of Physiology, Anatomy, Microbiology, La Trobe University, Melbourne, VIC 3083, Australia; 19 Department of Medicine and Immunology, Monash University, Melbourne, VIC 3800, Australia; 20 Department of Cardiometabolic Health, University of Melbourne, Parkville, VIC 3010, Australia; 21 Instituto de Investigación Sanitaria de la Fundación Jiménez Díaz UAM, 28040 Madrid, Spain; aortiz@fjd.es; 22 Centre for Genomic and Experimental Medicine, Institute of Genetics and Cancer, University of Edinburgh, Edinburgh EH16 4SB, UK; archie.campbell@ed.ac.uk; 23 Department of Pathology, Cardiovascular Research Institute Maastricht (CARIM), University of Maastricht, 6211 Maastricht, The Netherlands; 24 Aachen-Maastricht Institute for Cardiorenal Disease (AMICARE), University Hospital RWTH Aachen, 52074 Aachen, Germany

**Keywords:** cardiovascular events, coronary artery disease, heart failure, chronic kidney disease, personalized medicine, urinary biomarkers

## Abstract

(1) Background: Kidney and cardiovascular diseases are responsible for a large fraction of population morbidity and mortality. Early, targeted, personalized intervention represents the ideal approach to cope with this challenge. Proteomic/peptidomic changes are largely responsible for the onset and progression of these diseases and should hold information about the optimal means of treatment and prevention. (2) Methods: We investigated the prediction of renal or cardiovascular events using previously defined urinary peptidomic classifiers CKD273, HF2, and CAD160 in a cohort of 5585 subjects, in a retrospective study. (3) Results: We have demonstrated a highly significant prediction of events, with an HR of 2.59, 1.71, and 4.12 for HF, CAD, and CKD, respectively. We applied in silico treatment, implementing on each patient’s urinary profile changes to the classifiers corresponding to exactly defined peptide abundance changes, following commonly used interventions (MRA, SGLT2i, DPP4i, ARB, GLP1RA, olive oil, and exercise), as defined in previous studies. Applying the proteomic classifiers after the in silico treatment indicated the individual benefits of specific interventions on a personalized level. (4) Conclusions: The in silico evaluation may provide information on the future impact of specific drugs and interventions on endpoints, opening the door to a precision-based medicine approach. An investigation into the extent of the benefit of this approach in a prospective clinical trial is warranted.

## 1. Introduction

Cardiovascular diseases, including coronary artery disease (CAD) and heart failure (HF), along with chronic kidney disease (CKD), are the leading causes of morbidity and mortality worldwide [1,2]. These conditions place a significant burden on the affected individuals and healthcare systems globally. Efforts to reduce the known cardiovascular and kidney disease risk factors, such as hypertension, high cholesterol levels, a sedentary lifestyle, diabetes, obesity, and smoking, help to prevent disease progression in some patients [2,3]. Advances in medical care and novel treatments have improved the prognosis of individuals who are affected by these chronic diseases [1,4,5]. However, despite this progress, the factors associated with disease progression in individual patients are poorly understood. While traditional clinical risk factors and underlying molecular mechanisms can explain a significant part of the attributable risk [6,7], their predictive power for future cardiovascular or kidney events is limited, or has not been evaluated, and, in certain cases, may not be readily applicable in a clinical setting [7,8,9,10,11]. 

Furthermore, CAD, HF, and CKD require a complex treatment regimen comprising multiple drug combinations. Randomized trials have demonstrated the value of different individual treatments in preventing future cardiac or kidney events, reducing mortality, and managing symptoms [12,13,14,15,16]. However, the benefits of such treatments are only detected in some patients, and a substantial number of individuals still progress to terminal organ failure, despite the treatment. The commonly recommended treatments include lifestyle interventions, including dietary changes, antiplatelet therapy, β-blockers, angiotensin-converting enzyme inhibitors (ACEI), angiotensin receptor blockers (ARB), mineralocorticoid receptor antagonists (MRAs), glucagon-like peptide-1 receptor agonists (GLP1 RAs), dipeptidyl peptidase-4 inhibitors (DPP4i), and sodium-glucose co-transporter 2 inhibitors (SGLT2i) [17,18]. However, while these drugs demonstrably have an impact on the notional targets, such as the reduction in blood pressure or blood glucose, the targets are often surrogates for the real reason to treat—that is, preventing (or delaying) end-organ damage. That is more difficult to assess and needs a much longer time scale than days or weeks. There are currently no methods to predict treatment success in individuals or to give guidance on the optimal therapy for an individual patient. 

Recent advances in biomarker research have contributed to the development of predictive classifiers that are more accurate markers of the progression towards adverse outcomes, including severe disease or mortality [7,19,20]. Multidimensional urinary peptide profiles seem to be particularly promising for predicting the outcome at early stage and can show the effect of a treatment on different chronic diseases at a molecular level [7,11,21,22,23]. Particularly, in cancer research, the biomarker-based approach has made significant progress, from basic science to clinical validation [24,25]. For example, in diseases such as breast cancer, lung cancer, and melanoma, increasing attention has been directed towards genetic biomarkers being used as pivotal guides for treatment decisions [26,27].

To the best of our knowledge, no study has yet investigated the potential ability of biomarker-based information to predict the potential impact of different interventions in decreasing the risk of events (critical progression or death) from cardiovascular or kidney diseases on a personalized level. The objectives of this study were as follows: (1) to assess the efficacy of three previously developed urinary-peptide-based classifiers as biomarkers for predicting CAD, HF, or CKD events; and (2) to investigate the individual impact of prophylactic or therapeutic interventions in silico, with specific active agents, with the hypothesis that the treatment that shows the most pronounced effect in silico should be the optimal personalized therapeutic strategy.

## 2. Results

### 2.1. Clinical Characteristics of Population

A total of 5585 datasets were extracted from the database (Table 1). The baseline characteristics are shown in Table 2. 

### 2.2. Peptide-Based Classifiers and Prediction of Events

**Figure 1:**
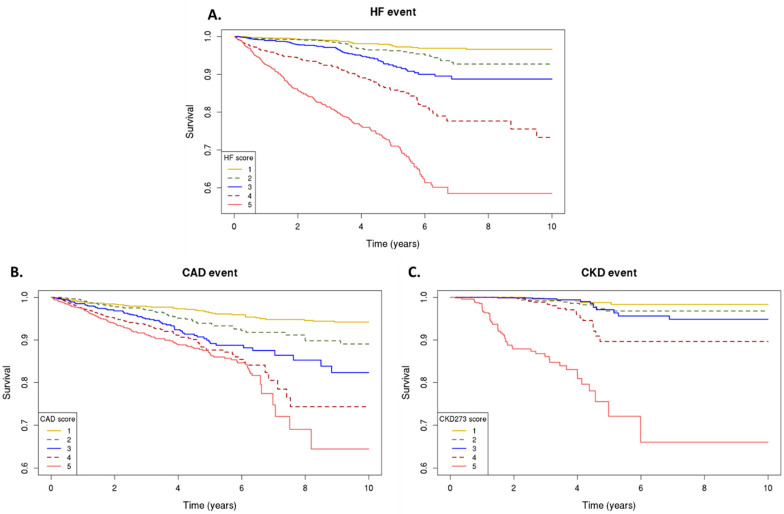
Urinary peptidomics classifiers and primary outcomes. Kaplan–Meier curves for the primary outcome; classifier scores from the lowest (1) to highest (5) quintile, for risk of heart failure events, as assessed by HF2 (**A**), coronary artery disease events, as assessed by CAD160 (**B**), and chronic kidney disease progression, as assessed by CKD273 (**C**).

The association between the classifiers and the risk of cardiovascular/kidney events is detailed in Table 3. The individuals were divided into quintiles with different relative risk, according to their classifier scores (Table S1). The event rates for the outcome of cardiovascular/kidney events varied across the five score subgroups. There was a stepwise increase in the risk of an adverse event with each quintile, that is, the individuals with higher classifier scores, as represented in the 5th quintile, had higher rates of the primary outcome compared to those individuals in the lower quintile of the classifier (1st quintile) (Figure 1). 

### 2.3. Personalized In Silico Prediction of Treatment Efficacy

**Figure 2:**
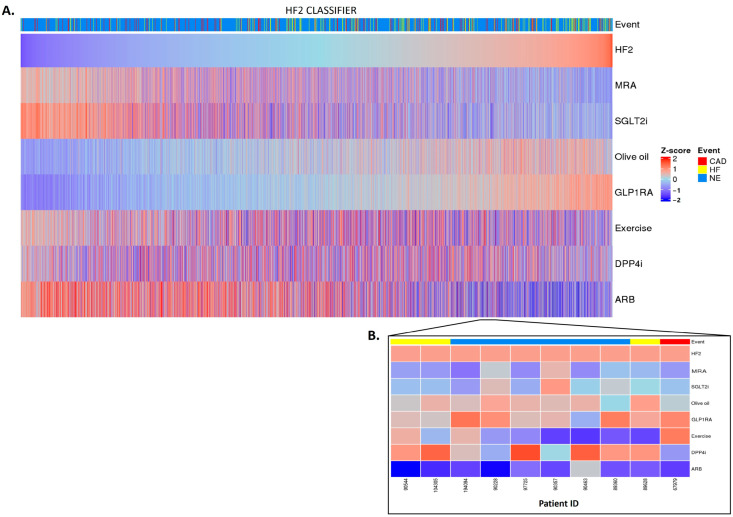
HF2 classifier treatment responses. HF2 scores were z-scaled across samples for visualization. Heatmap HF2 classifier treatment responses of 5585 patients (**A**). The top of the heatmap shows the event information. Samples (columns) were ordered based on the HF2 score prior to in silico treatment, from lower scores (left) to higher scores (right). The zoomed-in heatmap shows the treatment response of HF2 in 10 patients (**B**). Patients who were already receiving one of the treatments at the beginning of the study are depicted in gray.

**Figure 3:**
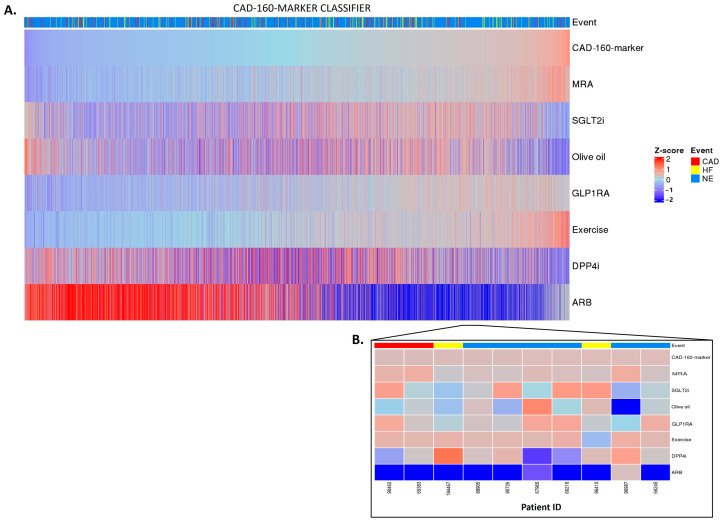
CAD-160-marker classifier treatment responses. CAD-160-marker scores were z-scaled across samples for visualization. Heatmap CAD-160-marker classifier treatment responses of 5585 patients (**A**). The top of the heatmap shows the event information. Samples (columns) were ordered based on the CAD-160-marker score prior to in silico treatment, from lower scores (left) to higher scores (right). The zoomed-in heatmap shows the treatment response of the CAD-160-marker classifier in 10 patients (**B**). Patients who were already receiving one of the treatments at the beginning of the study are depicted in gray.

**Figure 4:**
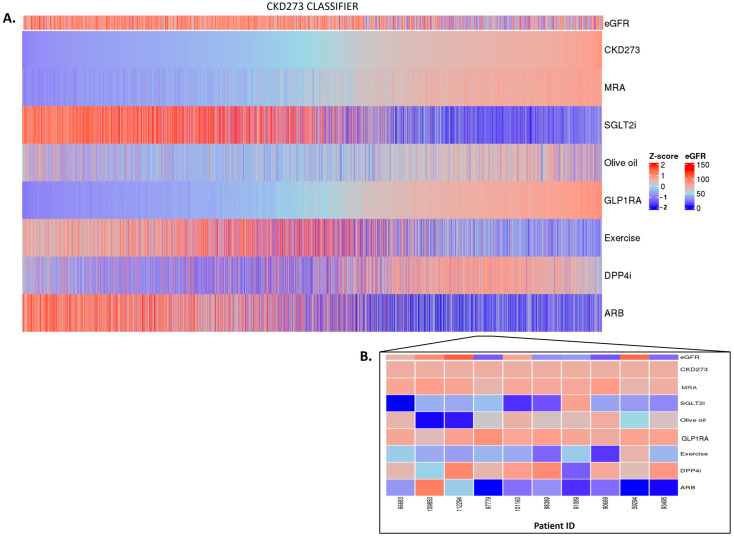
CKD273 classifier treatment responses. CKD273 scores were z-scaled across samples for visualization. Heatmap CKD273 classifier treatment responses of 5585 patients (**A**). The top of the heatmap shows the baseline eGFR value information. Samples (columns) were ordered based on the CKD273 score prior to in silico treatment, from lower scores (left) to higher scores (right). The zoomed heatmap shows the treatment response of the CKD273 classifier in 10 patients (**B**). Patients who were already receiving one of the treatments at the beginning of the study are depicted in gray.

Having established a highly significant association between the classifier scores and the outcomes, we investigated whether the in silico treatment effect (e.g., the adjustment of the peptide intensities based on the treatment response), as described in Section 4, had an impact on the classifiers. The in silico treatment had a significant impact on the classifiers, as shown in Figure 2, Figure 3 and Figure 4 (also shown in Table S1). The heatmap representation of the sorted scores before the in silico treatment revealed an alignment of HF events, CAD events, and CKD progression with higher scores (Figure 2, Figure 3 and Figure 4). This observation reinforces the predictive capability of the scores and their association with HF and CAD events and CKD progression.

After the in silico treatment, MRA, SGLT2i, and ARB treatments had a positive effect on the HF2 classifier in the individuals with higher scores, suggesting a potential beneficial impact of the treatment, especially in those with a higher baseline risk (and likely more advanced disease (Figure 2A)). The olive oil and GLP1R agonist treatments showed a positive impact mostly in the individuals at low risk of HF events. DPP4i and exercise had inconsistent effects across the different scores, making their impact on patients with a high risk of HF events less evident. The predictions showed individual differences in the treatment impact (Figure 2B).

Regarding the CAD-160-marker classifier, distinctly different treatment responses were observed when comparing the high-CAD-160-marker-score and low-CAD-160-marker-score groups (Figure 3A). Among the individuals with higher scores, the olive oil, DPP4i, and especially ARB treatments were predicted to present positive impacts, with the ARB treatment being notably effective for patients at high risk of CAD events. Nonetheless, the individual predictions displayed unique differences, emphasizing the personalized nature of the prediction of treatment response (Figure 3B).

In the context of CKD, no major impact was observed for spironolactone or for GLP1RA. In the patients with high CKD273 scoring, with many having eGFR values of less than 60 mL/min per 1.73 m^2^, the SGLT2i, olive oil, exercise, and ARB treatments exhibited treatment responses, with SGLT2i and ARB treatments showing a more pronounced impact. In contrast, the olive oil treatment seemed to have a positive impact mostly in the patients with lower scores (Figure 4A,B). The impact of DPP4i varies among patients, and, in certain cases, it demonstrates a positive effect in the advanced stages of the disease. 

## 3. Discussion

The identification of the biomarkers that aid physicians in decision making and treatment planning for patients with cardiovascular or kidney disease will serve a major clinical need. The early diagnosis of cardiovascular diseases and CKD is challenging, as the patients may remain asymptomatic in the early stages, leading to late-stage clinical presentations and diagnosis/detection. Additionally, considering the significant inter-individual variability in response to different treatments, uncertainty remains about how the development of an event can be best avoided, or at least delayed. While a substantial number of studies have demonstrated the potential value of biomarkers in predicting disease progression (renal as well as cardiovascular events [7,8,9,10,11]), these studies typically did not investigate the far more relevant topic (from the patient point-of-view) of the prediction of optimal intervention. The prediction of drug response on a population-based level was proposed by the group from Heerspink [28], but not on an individual level. Therefore, the crucial need for non-invasive biomarkers for early disease detection, and to understand the impact of the different treatments, becomes evident, enabling timely individual (personalized) treatment and the prevention of chronic disease progression, ultimately improving patients’ outcomes.

Multiple drugs are available that impact risk factors such as elevated blood pressure, blood glucose, or cholesterol. The normalization of these parameters can generally be easily and rapidly assessed. However, the question of whether the normalization of these parameters has an optimal desired beneficial impact on target organ damage on an individual level cannot be easily answered, and would require long-term follow-up, which is not compatible with clinical practice. What is needed is an approach to assess, and, ideally, even predict, the impact of the drug on the outcome on the target organ damage. In this study, we assessed three established urinary-peptide-based classifiers, HF2, CAD-160-marker, and CKD273 [20,29,30], designed to predict the risk of major complications or mortality in individuals at high risk of, or already suffering from, chronic cardiovascular or kidney disease conditions, and we investigated the potential impact of different interventions on reducing the occurrence of these events.

Personalized intervention was developed initially in oncology, where personalized treatment is now routinely applied, based on certain oncogenic mutations that can be targeted with specific drugs [24,25,26,27]. This approach has proven to be quite successful. A similar approach, targeting specific mutations, does not seem to be applicable in kidney and cardiovascular disease, as these are generally not driven by a specific genetic change, but rather by a number of different factors, some possibly being genetic-based, while most are the results of environmental impact. This fact has inspired the development of the following approach: instead of targeting a specific mutation, we aimed at “normalizing” multiple disease-associated changes. The approach was also inspired by the application of the Connectivity Map (CMap) [31,32,33], where potentially beneficial drugs are defined based on the “normalization” of a disease-specific transcriptome or proteome signature.

The study presented here has two main results. The first result is the demonstration of the prognostic value of the three applied urinary peptide classifiers in a very large cohort of >5000 subjects. This result further confirms the previous reports [20,29,30] in large cohorts. While such a prognosis is valuable in guiding the treatment and management, it obviously lacks specific guidance on the treatment. This fact leads to the second main result. Applying the previously established impact of the specific treatment on the urinary peptides allows for the implementation of the “in silico treatment”, which may be used to guide personalized intervention, based on the predicted response. Using this in silico approach, we have achieved individualized prediction of the effects of seven different treatment approaches based on the urinary-peptide-based classifiers. These findings offer a novel approach towards personalized treatment strategies and risk management for patients at risk of cardiovascular or kidney diseases, based on the predicted molecular impact of the specific treatment.

Previous studies have already demonstrated the predictive performance of the HF2 model, the CAD-160-marker model, and the CKD273 model, in different populations for the respective clinical conditions [20,29,30]. In our larger population, we observed effective risk stratification based on the model scores, successfully identifying the patients at higher risk of cardiovascular/kidney events. Specifically, the individuals in the lower-score group exhibited a reduced risk of HF, CAD, and CKD events compared to those in the higher-score group.

Regarding the individualized prediction of the treatment impact, we observed significant effects with interventions such as SGLT2i, ARB, MRA, DPP4i, and lifestyle changes. The overall observations are consistent with the results from previous intervention studies. Specifically, it appears that a benefit of ARB in CKD is most prominent in subjects with the highest risk, likely with late-stage disease, which is in agreement with the failure to demonstrate a significant benefit of ARB at the early of stage disease [34]. We have also detected a more pronounced benefit of SGLT2i, particularly in subjects with a high risk of HF and CKD, but to a much lesser degree in the context of CAD, which is in very good agreement with the respective intervention studies [35,36,37,38]. The impact, in the context of CAD, appeared to be most prominent for ARB in the subjects with an increased risk, which was also observed in the intervention trials [39,40]. MRA is a recommended treatment for HF in individuals with reduced and preserved ejection fraction. Notably, our findings have revealed a beneficial effect of MRA in HF among individuals at the highest risk, but not as clear in CAD or CKD, in line also with the results of the PRIORITY trial [11]. Our results are consistent with those of previous clinical trials, supporting the potential efficacy of MRA to improve HF outcomes [41]. However, the impact of MRA on CAD or CKD has not been clearly demonstrated. In CKD, MRA showed an early effect on renal function changes but did not have any longer-term effects [42]. 

Several studies have investigated the potential beneficial effects of GLP1RA and DPP4i on cardiovascular and kidney diseases [43,44,45,46]. Some clinical studies have suggested that these drugs may have a beneficial impact on the progression of these conditions; however, data from clinical trials remain somewhat controversial [47]. In our study, we observed a positive impact in individuals with a higher risk of CAD. However, further trials with appropriate power and design are necessary. Larger and well-controlled clinical trials will provide a clearer understanding of the potential benefits of these drugs.

Lifestyle modification is generally recommended for the management of cardiovascular and kidney diseases. However, when it comes to CKD and CAD, physical activity recommendations should be carefully considered, depending on the patient’s condition, due to the potential risk of impairing kidney function and increasing proteinuria, or triggering cardiovascular events during exercise [48,49]. As for olive oil in the diet, some evidence has suggested that this intervention may have a beneficial impact in preventing cardiovascular or kidney events [50,51]. Specifically, our observations have revealed that the impact of lifestyle intervention seems to have an individual pattern, with a positive impact in the patients at both low and high risk. Regarding exercise, we observed a positive impact in the individuals at lower risk of CAD events and in some individuals at high risk. Meanwhile, it seemed to have a preventive effect on HF and CKD events in the individuals at high risk, which is consistent with the findings from previous studies [52,53]. 

The study also has shortcomings, and the results should be interpreted with caution. First, this is a retrospective study based on data collected in the context of multiple different previous studies. However, the large number of subjects included is expected to counteract the potential bias introduced by some of the specific previous cohorts. Furthermore, the results observed are fully in line with the previous observations, further supporting the validity. Second, the impact of the treatment on the urinary proteome by the different means of intervention is not fully comparable, as the number of subjects in the previous studies, the demographic characteristics, and the duration of the intervention differed. To counteract these issues, the data were normalized to Z-scores to prevent the dominance of one specific intervention. Given the results, the fact that some types of intervention are predicted to be specifically beneficial in certain situations (e.g., SGLT2 inhibition indicating a benefit in most patients with a high risk of HF and CKD events, but not in subjects with a high risk of CAD events), and that this observation is in very good agreement with the observations in the intervention trials reported, further supports the validity of the approach. At the same time, inter-individual variability has been observed, which highlights the need for the personalized aspect of the presented in silico predictor to be further tested in the context of a prospective clinical intervention trial. Along the same lines, we have not formally demonstrated that the prediction of the best-suited intervention does, in fact, provide a significant benefit to the patients, with respect to preventing them from experiencing any of the patient-relevant endpoints. Such a benefit can only be demonstrated in a prospective trial. However, based on the data available, we feel that using this approach may well be justified in a situation when guidance on the ideal intervention is missing. In addition, we are currently in the process of planning and initiating a prospective trial that is expected to demonstrate a significant benefit.

## 4. Materials and Methods

### 4.1. Study Participants and Study Design 

This study included 5585 datasets from the following previous studies: PRIORITY, DIRECT, FLEMENGHO, CACTI, CardioRen, CAD prediction, Generation Scotland, HOMAGE, SUNmacro, and UZ-Gent. Detailed information on the designs and methods used in these studies are available in the previous publications [11,30,54,55,56,57,58,59,60,61,62,63,64,65]. The inclusion criteria were as follows: availability of estimated glomerular filtration rate (eGFR, calculated using the CKD Epidemiology Collaboration (CKD-EPI) formula), information on cardiovascular events, and availability of follow-up information. The endpoints were defined as follows: For coronary artery disease, the event was defined as non-fatal and fatal acute myocardial infarction. A heart failure event was defined as hospitalization or death from heart failure. For CKD, an event was defined as a decline of ≥40% in eGFR values during the follow-up, and the date when this decline was observed was considered as the duration of the follow-up. Only one (the first) endpoint per patient was allowed, and if an endpoint was reached, the further endpoints were censored. 

All individuals with urine samples at the baseline visit were included in the analysis. Several covariables, including body mass index, age, sex, blood pressure, and eGFR, were determined at the time of the baseline assessment. The median follow-up was 3.74 ± 3.36 years. The study was conducted according to the guidelines of the Declaration of Helsinki and all datasets were fully anonymized. This study was approved by the ethics committee of the Hannover Medical School, Germany, under the reference number 3116-2016.

### 4.2. Peptide-Based Classifiers and Prediction of Events

The classifier CKD273 was used for the prediction of CKD events and the impact of the treatments [20]. The predictive capacity of, and the impact of treatments on, the classifiers HF2 and CAD-160-marker, were assessed for HF and CAD, respectively [29,30]. The scores for each classifier were calculated using a support vector machine (SVM) algorithm, integrated into the MosaCluster software. All statistical tests were performed in R statistical software (R version 4.1.0, R Foundation for Statistical Computing, Vienna, Austria). The Kaplan–Meier estimator was applied to assess the association of longitudinal survival with each classifier. Corresponding hazard ratios (HR) were estimated using Cox regression models, and log-rank tests were used to assess the hypothesis of no group differences in hazard functions. All survival analyses were carried out using the R package “survival”.

### 4.3. In Silico Impact of Treatments

**Figure 5:**
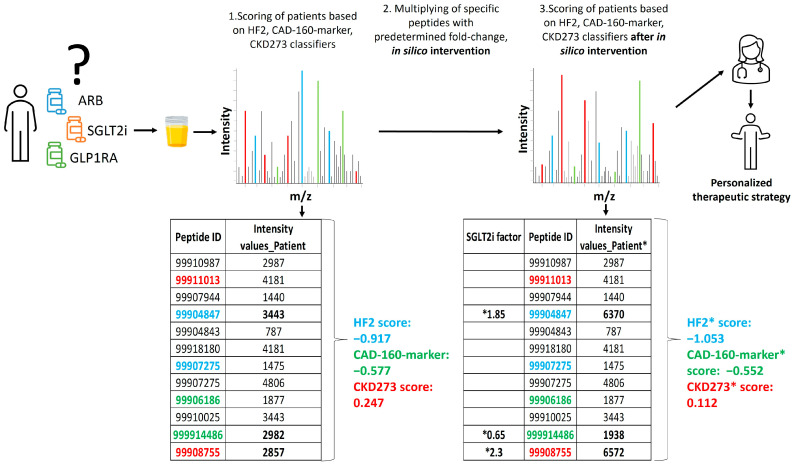
Schematic depiction of the study design. The relative abundance of 5071 sequenced urinary peptides was investigated using CE-MS. Data on some selected peptides (ID) for 1 subject are shown. Several of these peptides were previously identified as being associated with the respective pathophysiology and combined into classifiers CKD273, CAD160, and HF2. Some of these peptides are labelled with their respective color. In the first step, the patient received a score for progression to event using the predefined urinary classifiers. Of the peptides shown, 3, labelled in bold, were found to be affected by the SGLT2i treatment. In the second step, the abundance of these 3 peptides was adjusted, based on the observed fold change, as a result of the treatment (“in silico treatment”). The classifier score was then re-calculated (labelled *), and the result was compared to the initial scoring, where a decrease in the scoring indicated the benefit of the treatment. In this example, the relevant impact of the SGLT2i treatment on a CKD and HF event is predicted, but not the impact on CAD.

To assess the impact of various treatments on the classifiers for CAD, HF, and CKD, the impact on the urinary peptidomic profiles from five different drug-based interventions (MRA, SGLT2i, GLP1RA, DPP4i, and ARB), one dietary intervention (olive oil), or from exercise were applied. These data were generated in previous studies that were either published, submitted for publication, or unpublished (exercise). Briefly, the effect of the interventions on the urinary peptidomic profiles was assessed, and the fold change values (as a result of the intervention) were determined. To predict the impact of the treatment, these fold changes were then used to multiply the intensities of the respective peptides in each patient, and the predictor (CKD273, HF2, or CAD-160-marker) scores were re-calculated. A decrease in the classifier score was indicative of a positive impact of the treatment on the outcome, as depicted in Figure 5. The results were visually represented using heatmaps generated with the R package “ComplexHeatmap”. 

## 5. Conclusions

In conclusion, this study pioneers a groundbreaking in silico approach to predicting the impact of different drugs on an individual’s urinary peptidomic signature and may provide information on the future impact of specific drugs on hard endpoints in that individual, opening the door to a precision-based medicine approach to selecting the optimal treatment for individuals with, or at risk of, CAD, HF, or CKD progression. While previous studies have demonstrated the potential value of biomarkers in predicting disease progression and related events, the focus has primarily been on prognosis rather than on guiding optimal interventions. This study aims to fill this critical gap. By assessing the previously established effects of the specific treatments on urinary peptides, this approach enables in silico treatment and offers personalized predictions of treatment impact. The results show the significant effects of different interventions, such as SGLT2i, ARB, MRA, DPP4i, and lifestyle changes, based on individualized risk profiles. 

These findings open the door to a new era of personalized treatment strategies and risk management, offering a pathway to improved outcomes for patients at risk of cardiovascular or kidney diseases. The robustness of the results and their consistency with previous observations lend credibility to the findings. Nevertheless, the performance of this in silico test should be validated in a prospective clinical trial as a critical next step to definitively confirm its clinical utility. 

## Figures and Tables

**Table 1:** Summary of the studies included in the analysis.

Characteristic	N = 5585	Study Information
Study		
CACTI	19 (0.34%)	Adults with type 1 diabetes
CADPredictions	147 (2.63%)	Adults with acute coronary syndromes
CardioRen	116 (2.08%)	Adults with heart failure with reduced ejection fraction
DIRECT	769 (13.77%)	Adults with type 2 diabetes with normoalbuminuria
EPOGH	826 (14.79%)	Adults with type 2 diabetes treated with basal insulin
FLEMENGHO	65 (1.16%)	General population
Generation Scotland	450 (8.06%)	Adults with and without coronary artery disease (CAD)
HOMAGE	354 (6.34%)	Adults with heart failure
Predictions Groningen_Prag	38 (0.68%)	Adults with type 2 diabetes
PRIORITY	1761 (31.53%)	Adults with type 2 diabetes, normal urinary albumin excretion, and preserved renal function
SUNmacro	580 (10.38%)	Adults with type 2 diabetic nephropathy
UZ-Gent	460 (8.24%)	Adults with chronic kidney disease
n (%)		

**Table 2:** Baseline characteristics of study participants.

Characteristic	N = 5585
Duration of follow-up (years)	3.75 (0.38, 7.11)
Clinic characteristics	
Age	62 (28, 82.3)
Female	3410 (61.06%)
sBP (mm Hg)	133 (105, 171)
dBP (mm Hg)	79 (58, 98)
Hypertension	2381 (42.63%)
Diabetes	3330 (59.62%)
eGFR (mL/min/1.73 m^2^)	81.89 (23, 117)
BMI (kg/m^2^)	28.4 (19.8, 41)
Urinary-peptide-based classifiers	
HF2	−0.29 (−0.76, 0.21)
CAD 160-marker	−0.32 (−0.71, 0.03)
CKD273	−0.50 (−0.84, −0.01)
Median (95% IC); n (%)	

Abbreviations: eGFR, estimated glomerular filtration rate (mL/min per 1.73 m^2^); BMI, body mass index; sBP, systolic blood pressure; dBP, diastolic blood pressure.

**Table 3:** Risk of HF events, CAD events, and CKD outcomes by baseline urinary peptidomics classifiers.

	Events/At Risk (%)	Model Unadjusted		Model (Adjusted for Age, BP, BMI, Sex, and eGFR)	
HF2	HF Events	HR (95% CI)	*p*-Value	HR (95% CI)	*p*-Value
Per 1-SD increment	472/5200 (9.08)	2.59 ± 0.047	<2 × 10^−16^	1.64 ± 0.056	1.72 × 10^−18^
Quintile 1	25/1041 (2.40)	Reference	Reference	Reference	Reference
Quintile 2	38/1040 (3.65)	1.81 ± 0.26	0.02	1.15 ± 0.26	0.60
Quintile 3	59/1040 (5.67)	3.17 ± 0.24	1.42 × 10^−6^	1.51 ± 0.24	0.09
Quintile 4	119/1040 (11.44)	7.21 ± 0.22	4.60 × 10^−19^	2.53 ± 0.23	5.92 × 10^−5^
Quintile 5	231/1039 (22.23)	16.20 ± 0.21	3.15 × 10^−39^	3.84 ± 0.23	5.64 × 10^−9^
**CAD-160-marker**	**CAD events**				
Per 1-SD increment	384/5112	1.72 ± 0.050	<2 × 10^−16^	1.33 ± 0.057	5.55 × 10^−7^
Quintile 1	46/1024 (4.49)	Reference	Reference	Reference	Reference
Quintile 2	55/1020 (5.39)	1.84 ± 0.20	2.45 × 10^−3^	1.39 ± 0.20	0.11
Quintile 3	71/1026 (6.92)	2.93 ± 0.19	2.77 × 10^−8^	2.13 ± 0.20	1.25 × 10^−4^
Quintile 4	91/1019 (8.90)	3.92 ± 0.19	2.65 × 10^−13^	2.53 ± 0.19	1.32 × 10^−6^
Quintile 5	121/1023 (11.83)	4.73 ± 0.18	4.93 × 10^−18^	2.82 ± 0.19	3.32 × 10^−8^
**CKD273**	**CKD events**				
Per 1-SD increment	113/3635 (3.11)	4.19 ± 0.094	<2 × 10^−16^	3.18 ± 0.121	1.03 × 10^−21^
Quintile 1	6/732 (0.82)	Reference	Reference	Reference	Reference
Quintile 2	11/722 (1.52)	2.02 ± 0.51	0.17	1.96 ± 0.50	0.18
Quintile 3	11/730 (1.51)	2.13 ± 0.51	0.14	1.80 ± 0.51	0.25
Quintile 4	22/724 (3.04)	5.58 ± 0.46	2.07 × 10^−4^	5.33 ± 0.47	3.55 × 10^−4^
Quintile 5	63/727 (8.67)	35.47 ± 0.43	1.61 × 10^−16^	19.59 ± 0.47	7.32 × 10^−11^

## Data Availability

Data is contained within the article and Supplementary Material.

## Supplementary Materials

The following supporting information can be downloaded at: https://www.mdpi.com/article/10.3390/ph16091298/s1, Table S1: Classifier score before and after the in silico treatment.
